# Severe Hypertriglyceridemia-Induced Necrotizing Pancreatitis Associated With Ketogenic Diet in a Well-Controlled Patient With Type 2 Diabetes Mellitus

**DOI:** 10.7759/cureus.20879

**Published:** 2022-01-02

**Authors:** Jacqueline T Chan, Pooja J Mude, Whitney Canfield, Jinal Makhija, John Erikson L Yap

**Affiliations:** 1 Pediatric Endocrinology, Augusta University Medical College of Georgia, Augusta, USA; 2 Internal Medicine, Augusta University Medical College of Georgia, Augusta, USA; 3 Dietetics, Augusta University Medical College of Georgia, Augusta, USA; 4 Infectious Diseases, Rush University Medical Center, Chicago, USA; 5 Gastroenterology and Hepatology, Augusta University Medical College of Georgia, Augusta, USA

**Keywords:** hypertriglyceridemia-induced acute pancreatitis, carbohydrate-restricted diet, diabetes type 2, triglyceride, severe pancreatitis, keto diet

## Abstract

The ketogenic diet (keto diet) has become an increasingly popular approach for both weight loss and as an alternative diet for type 2 diabetes mellitus (T2DM). Owing to the nature of the keto diet, patients are at risk of developing hypertriglyceridemia (HTG) due to the high amount of triglycerides consumed by individuals during the initiation of this diet. Acute pancreatitis can result from HTG. We present a case of a 19-year-old African American male with well-controlled T2DM and no history of HTG who developed severe necrotizing HTG-induced pancreatitis after an unsupervised three-month trial of the keto diet.

## Introduction

The concept of the ketogenic diet (keto diet) started in 1911 when fasting was first used as a treatment for epilepsy. The term ketogenic emerged almost a decade later in light of two important observations. The first one was that normal subjects under starvation or consuming diets with extremely low portions of carbohydrates and extremely high fat would produce acetone and beta-hydroxybutyric acid. The second was that the benefits of fasting could be obtained if ketonemia was produced [1]. Media has helped the popularization of several types of diets, including the keto diet. 

Type 2 diabetes mellitus (T2DM) is mainly controlled via medical nutrition therapy, which involves controlling glycemia by limiting carbohydrate intake [2]. Currently, there are many claims that the keto diet, whether it be a classic ketogenic diet plan or an alternative, helps control blood sugar, has led to weight loss, and can "cure" T2DM in individuals [3]. There is now a large market of keto products that are readily available, making it easy for any individual to embark on a keto diet regardless of the presence or absence of medical advice. Unfortunately, the keto diet can contribute to the worsening of hypertriglyceridemia (HTG) if not properly monitored. HTG is an uncommon but well-established etiology of acute pancreatitis and the risk and severity of acute pancreatitis increase with higher triglyceride levels (>1000 mg/dL) [4,5]. HTG is not an obligatory component of diabetes mellitus but is often found in uncontrolled diabetes mellitus.

There are only a handful of reports in the literature that describe HTG-induced pancreatitis due to the keto diet. We present a case of a patient with well-controlled T2DM who developed severe necrotizing HTG-induced pancreatitis after a trial of the keto diet.

This article was previously presented as a meeting abstract at the 2020 American College of Gastroenterology Meeting in October 2020.

## Case presentation

A 19-year-old male with no previous history of hyperlipidemia, class 3 obesity (BMI of 41), and well-controlled, insulin-dependent T2DM (HbA1c of 5.6%) self-initiated an unspecified keto diet with the goal of losing weight before going to college. His last physical exam revealed normal vital signs with a blood pressure of 117/76. His previous annual labs revealed a normal lipid profile, complete blood count, complete metabolic panel, and urine microalbumin. The patient was well-versed with his insulin to carbohydrate ratio and sliding scale blood sugar correction; he had excellent A1c levels for several years prior to admission. Unfortunately, his outpatient endocrinology follow-up was affected by the COVID-19 pandemic and his appointment was rescheduled given his long history of being a well-controlled diabetic. He was admitted to the ICU for severe acute pancreatitis after presenting with a one-day history of severe abdominal pain and vomiting.

**Figure 1:**
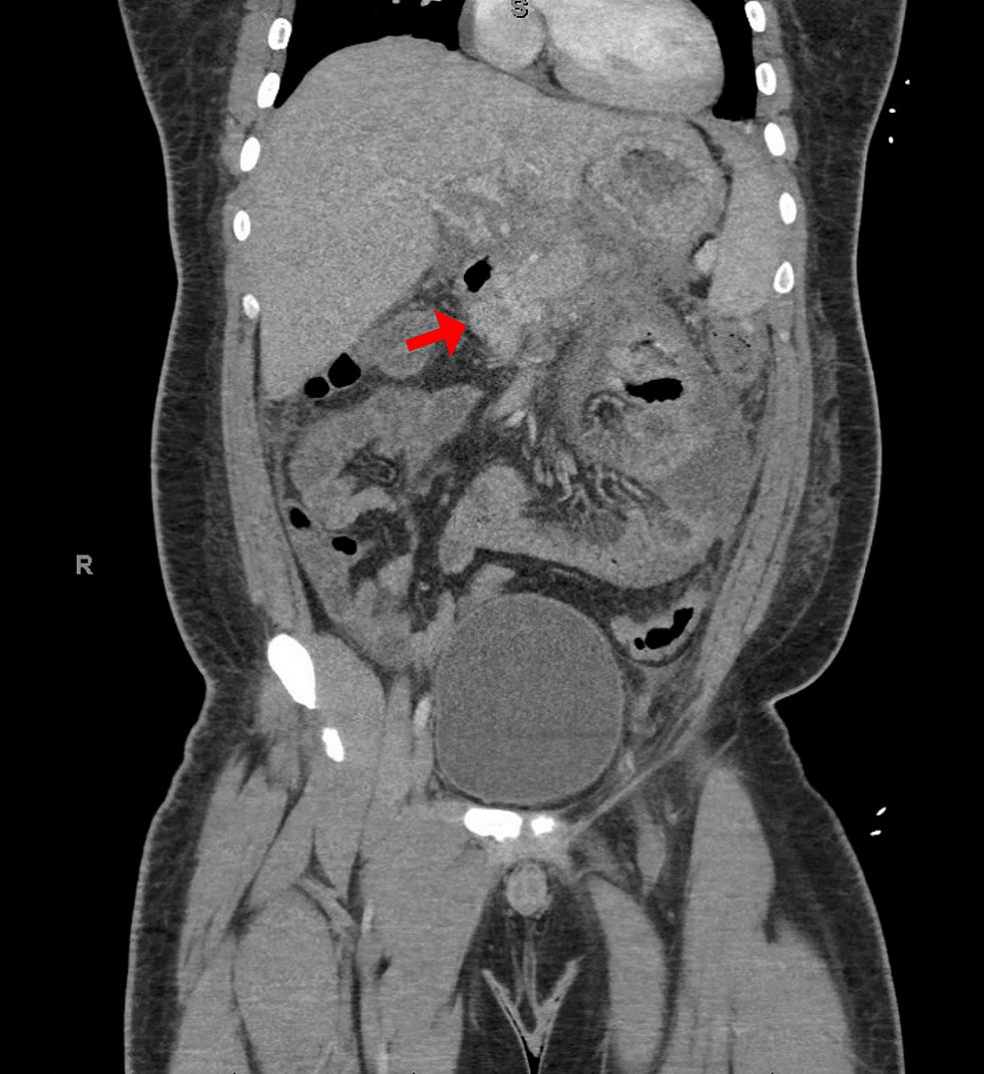
Acute pancreatitis demonstrating inflamed head of the pancreas with necrotic pancreatic tail

The patient stated he lost twenty pounds through the keto diet since starting it three months prior to presentation. He self-initiated his keto diet regimen through books and the Internet, and only consumed food labeled “ketogenic-friendly”. He drastically decreased his carbohydrate intake as well. The only medication the patient was on at this time was insulin, which he appropriately titrated down as he was consuming fewer carbohydrates. The patient’s home glucose measurements were consistently below 100mg/dL with no hypoglycemic episodes, consistent with his normal HbA1c of 5.6%. He denied alcohol use or any illicit drug use. His labs were pertinent for a lipase level of 2,195 U/L, serum glucose of 319 mg/dL without acidosis, and serum triglyceride level of 6,021 mg/dL. This elevated glucose was thought to be reactive to the stress of acute pancreatitis. He was appropriately managed with IV fluids, IV insulin drip, electrolyte replacement, fibrates, and statin, and eventually required apheresis. His acute pancreatitis progressed in severity and the patient ultimately developed respiratory failure requiring intubation, persistent fevers requiring IV antibiotics, and shock necessitating pressor support. Blood and urine cultures were negative. Imaging showed necrotic parenchyma involving the pancreatic body with large ascites concerning for pancreatic ascites and no gallstones (Figure 1). Paracentesis confirmed the diagnosis of pancreatic ascites with an amylase level of 25,389 U/L. He underwent an endoscopic retrograde cholangiopancreatogram (ERCP) for a pancreatic duct stent placement due to a pancreatic duct disruption causing pancreatic ascites. After a prolonged eight-week hospital course, he was discharged in stable condition. The patient was educated regarding the risks of HTG developing from an unsupervised keto diet and had close endocrinology and nutrition follow-ups. 

## Discussion

The keto diet comprises high fat, low carbohydrate, and adequate protein. The aim is to have the body use fat for energy instead of carbohydrates. There are four main ketogenic diet therapies: the classic keto diet (four: one, three: one ratios), the modified Atkins diet (MAD), the medium-chain triglyceride (MCT) oil diet, and the low glycemic index treatment (LGIT) diet. The MAD, MCT, and LGIT diets are usually a lower ratio of one:one or two:one, meaning one gram of net carbohydrates plus protein to one to two grams of fat [6]. The initial therapeutic benefits of the keto diet have been shown through many studies and randomized controlled trials and have demonstrated efficacy with seizure control [7]. In recent years, the keto diet has gained worldwide recognition as a tool to promote weight loss [8]. Due to the nature of the keto diet, it puts patients at risk of having HTG since the predominant form (98%) of dietary fat intake is in the form of triglycerides. Reduction in carbohydrate intake stimulates lipolysis, which then accelerates the breakdown of adipose tissue resulting in weight loss. The goal of the keto diet is to switch the preferred energy source of the body from available carbohydrates to stored triglycerides. Inadvertent intake of carbohydrates beyond the recommendation levels will switch back this preferred energy source to carbohydrates and not utilize the available triglycerides, resulting in high triglyceride levels [9]. 

HTG is an uncommon cause of acute pancreatitis (accounting for only 5-9% of all cases of acute pancreatitis) but is associated with higher rates of morbidity and complications, including persistent organ failure, when compared to other etiologies of acute pancreatitis [4,10]. One of the requirements for the diagnosis of HTG-induced pancreatitis is a triglyceride level of above 1,000mg/dL. The risk of developing acute pancreatitis increases to 5% with levels above 1,000mg/dL and 10-20% with levels beyond 2,000mg/dL [4]. The majority of patients with HTG-induced pancreatitis are younger males having comorbidities, such as T2DM and obesity, in contrast to those without HTG [5]. The causes of HTG-induced pancreatitis could be primary, such as through genetic disease, or secondary like our patient who developed HTG-induced pancreatitis after initiating keto diet. An increase in dietary triglyceride intake as part of the keto diet leads to an increased generation of free fatty acids (FFA) following the breakdown of circulating triglycerides by pancreatic lipase [11]. Lipotoxicity results from the increased accumulation of FFA, which then stimulates an inflammatory response causing the release of intracellular calcium and pancreatic acinar necrosis. This leads to pancreatic auto-digestion and acute pancreatitis [4].

Patients with HTG-induced pancreatitis present similarly to others with acute pancreatitis from other causes and manifest symptoms such as nausea, vomiting, and severe abdominal pain [8]. Physical examination may be pertinent for xanthomas on the body’s extensor surfaces and hepatosplenomegaly [4]. Diagnosing HTG-induced pancreatitis is comparable to that of acute pancreatitis from other etiologies, which requires fulfilling two out of the three criteria, namely: acute epigastric pain radiating to the back, elevated serum lipase at least three times the upper limit of normal, and characteristic imaging findings of acute pancreatitis plus an elevated triglyceride level of at least 1,000mg/dL [4,12]. While the clinical course of HTG-induced pancreatitis is the same as that of other causes of acute pancreatitis, it is associated with higher rates of morbidity including end-organ failure, infection, and shock, as well as higher rates of mortality [13].

The initial management of HTG-induced pancreatitis is similar to that of other causes of acute pancreatitis and involves aggressive intravenous hydration, initial bowel rest followed by early enteral nutrition, and symptomatic care with pain management [13]. Currently, there is no established guideline for the treatment of HTG-induced pancreatitis. Nonetheless, insulin, heparin, apheresis, plasmapheresis, and medications, including fibrates and omega-3 fatty acids, have been utilized to lower the serum triglycerides level of patients with HTG-induced pancreatitis with a goal of <500mg/L. This is correlated with clinical improvement [13].

Despite evidence of the positive effects of the keto diet by reducing carbohydrate intake resulting in lower body weight and better glycemic control in patients with T2DM, long-term safety and efficacy are still lacking. A review done by Bolla et al. had shown that pediatric patients who followed the keto diet and who are prone to ketosis, such as those with type 1 diabetes, and often had negative growth effects [14]. Care must be taken to ensure healthy dietary fat intake among patients interested in starting keto diets by working closely with a dietitian. If not done correctly, keto diets can have deleterious effects on the body and may include hypoglycemia, dehydration, renal calculi formation, and hyperlipidemia, particularly HTG-induced acute pancreatitis [8]. We only found one case involving a pediatric nine-year-old girl, with no known history of diabetes mellitus, who died of HTG-induced pancreatitis attributed to the keto diet [15]. Our case, to the best of our knowledge, is the first adult patient without a prior history of hyperlipidemia who developed HTG-induced acute pancreatitis after initiating a keto diet three months prior to presentation to promote weight loss. This case highlights the importance of recognizing the detrimental effects of keto diets and their role on serum triglycerides as a precipitating factor for HTG-induced acute pancreatitis. There are only a handful of reports in the literature that describe HTG-induced pancreatitis due to the keto diet, but patients and medical practitioners should be aware of the potentially dangerous relationship between the keto diet and pancreatic function even amongst patients with no history of hyperlipidemia.

## Conclusions

As keto diet grows in popularity for weight loss and as an alternative diet for diseases such as T2DM, awareness of its adverse effects should also be stressed and monitored. Patients and medical providers should take caution to avoid unnecessary risk while taking advantage of the benefits this diet provides. Specifically for patients with T2DM, the increased consumption of triglycerides can lead to HTG and predispose patients to develop a severe form of pancreatitis (HTG-induced severe pancreatitis) causing additional harm to our patients. Appropriate guidance by a registered nutritionist or dietician is necessary for patients who would like to take advantage of the keto-diet. Further studies on how to optimally monitor and incorporate the keto diet in this specific population are warranted.
